# Advances in Preparation and Biomedical Applications of Sodium Alginate-Based Electrospun Nanofibers

**DOI:** 10.3390/gels11090704

**Published:** 2025-09-03

**Authors:** Xuan Zhou, Yudong Wang, Changchun Ji

**Affiliations:** College of Biological and Chemical Engineering, Guangxi University of Science and Technology, Liuzhou 545006, China; 20230402010@stdmail.gxust.edu.cn

**Keywords:** electrospinning, sodium alginate, biomedical, tissue engineering, wound dressing

## Abstract

Sodium alginate (SA) has the advantages of good biocompatibility, water absorption, oxygen permeability, non-toxicity, and film-forming properties. SA is compounded with other materials to formulate a spinning solution. Subsequently, electrospinning is employed to fabricate nanofiber membranes. These membranes undergo cross-linking modification or hydrogel composite functionalization, yielding nanofiber composites exhibiting essential properties, including biodegradability, biocompatibility, low immunogenicity, and antimicrobial activity. Consequently, these functionalized composites are widely utilized in tissue engineering, regenerative engineering, biological scaffolds, and drug delivery systems, among other biomedical applications. This work reviews the sources, characteristics, and electrospinning preparation methods of SA, with a focus on the application and research status of SA composite nanofibers in tissue engineering scaffolds, wound dressings, drug delivery, and other fields. It can be concluded that SA electrospun nanofibers have great development potential and application prospects in biomedicine, which could better meet the increasingly complex and diverse needs of tissue or wound healing. At the same time, the future development trend of SA composite nanofibers was prospected in order to provide some theoretical reference for the development of biomedical textiles and to promote its development in the direction of being green, safe, and efficient.

## 1. Introduction

As the largest organ of the human body, the skin plays an important role in protection, sensation, and regulation. The healing process of skin is divided into four key stages, which are hemostasis, inflammation, proliferation, and remodeling [1,2,3]. However, severe trauma such as deep burn, diabetic ulcer, or chronic inflammation often lead to skin healing disorders, which are characterized by high risk of infection, excessive scar hyperplasia, or incomplete functional recovery [4,5,6]. Although traditional gauze dressing or antibiotics can alleviate symptoms, it is difficult to achieve efficient repair and functional reconstruction of the skin, and it is easy to adhere to the wound during replacement, causing secondary damage [7,8]. In the biomedical field, microorganisms such as bacteria may contaminate the surface of medical devices and implants, leading to wound infection and tissue inflammation, which increases the difficulty of treatment. In addition, bacterial clumps and extracellular matrices may form biofilms on the surface of medical devices, making it difficult for disinfectants such as antibiotics to penetrate, thus providing protection for bacteria and increasing the difficulty and cost of treatment. Previous studies have confirmed that the nanofiber composite membrane made of biocompatible and degradable materials could be used in the biomedical field, which could be applied to the wound tissue to achieve the effect of rapid healing without causing infection [9,10], and could also be made into tissue engineering scaffolds to provide sufficient space for the growth and proliferation of new cells [11].

SA, as a natural polysaccharide [12,13], is widely used because of its good biocompatibility [14], water absorption, oxygen permeability, non-toxicity, and film-forming properties [15,16,17,18,19]. Currently, there are various technologies for preparing alginate-based nanomaterials, such as electrospinning [20], electrostatic spraying [21], self-assembly [22], 3D printing [23], and micro jet spinning [24], etc. Among the above methods, electrospinning technology is a cutting-edge, convenient, economic, and commonly used method for the preparation of nanofibers [25]. Under the fine control of the spinning process, the performance and microstructure of nanofibers can be precisely customized by changing the fiber structure, operating temperature, solution conductivity properties, spinning solution concentration, and other key parameters so as to meet the diversified application needs [26,27,28,29]. There are extensive hydrogen bonds and intermolecular forces in the structure of alginate that prevent the necessary bonding between the molecular chains of alginate to produce uniform and continuous fibers. Electrospinning pure SA from aqueous solutions remains difficult under conventional conditions due to its polyelectrolyte nature and insufficient chain entanglement. However, certain modifications such as adding strong polar co-solvents like glycerol [30] or blending with spinnable polymers such as PEO or PVA can enable the formation of SA-based fibers. According to different application requirements to obtain nanofiber membranes with good fiber uniformity, strong mechanical properties and good degradation efficiency [31,32,33].

In addition, by adjusting the fiber diameter and orientation to simulate the topology of the natural extracellular matrix (ECM), cell adhesion and directional migration could be significantly improved for biomedical applications. Najafiasl et al. [34] prepared a wound dressing containing dexpanthenol (Dex) by electrospinning with polyvinyl alcohol (PVA)/sodium alginate (SA) and chitosan as the core–shell. In vivo studies of wound dressings containing Dex have shown better healing effects compared to wound dressings without Dex, demonstrating its potential in wound dressings. Khan et al. [35] mixed zinc oxide (ZnO) nanoparticles and oregano oil into poly (L-propylcaprolactone CO)-ε-caprolactone) (PLCL) core–shell nanofibers, which showed good antibacterial activity and accelerated wound healing. Alginate electrospun nanofiber materials have the characteristics of high specific surface area, high drug loading capacity, controllable drug delivery, small side effects, and biodegradability and are widely used in biomedical fields such as tissue stents, drug delivery, and medical dressings [36,37,38].

**Figure 1:**
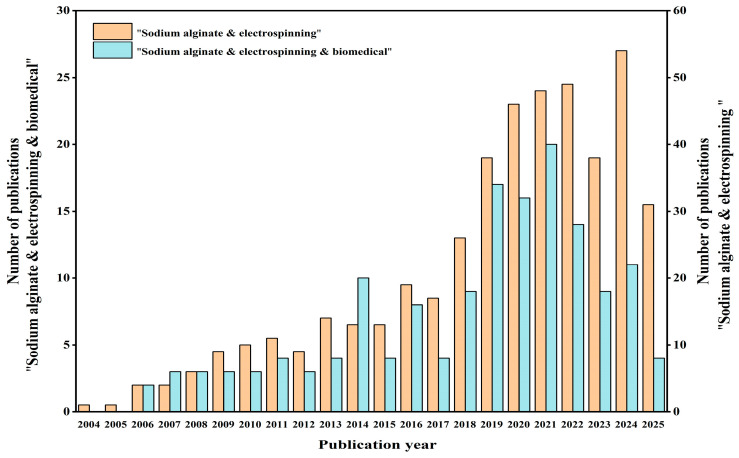
Statistics of the literature retrieval on the “Web of Science” platform with the all fields of “sodium alginate & electrospinning” and “sodium alginate & electrospinning & biomedical”.

The literature on “sodium alginate & electrospinning” and “sodium alginate & electrospinning & biomedical” was smart-searched on the “Web of Science” platform from 2004 to 2025. Figure 1 illustrates a yearly growth in the literature pertaining to “sodium alginate & electrospinning”. Despite the limited quantity of research papers focusing on “sodium alginate & electrospinning & biomedical”, there is an increasing trend, suggesting a promising future for electrospun nanofibers in biomedical research.

Recently, there has been a gradual increase in reviews related to alginate [39,40,41]. However, there is currently a lack of systematic summaries of the latest developments in sodium alginate electrospinning in the biomedical field. In addition, there is relatively little mention of how the monomer ratio of sodium alginate affects biomedical functions in existing reviews. Therefore, in this paper, the sources, monomer proportional performance, and electrospinning methods of alginate were introduced and the latest research progress of alginate electrospun nanofibers in tissue engineering scaffolds, wound dressings, drug release, and other aspects were reviewed in detail. Finally, the challenges and future research directions of alginate electrospun nanofibers in the biomedical field were summarized and prospected. For the literature review, we mainly applied “sodium alginate electrospinning” as the central keyword, and searched the relevant literature on databases including Web of Science, Scopus, and ScienceDirect; then, using additional keywords such as “biomedical”, “tissue engineering”, “wound dressing”, and “drug delivery”, the related articles were screened and summarized mainly from the past five years.

## 2. Sodium Alginate

Alginate exists in the cell matrix of brown algae in the form of ionic cross-linking gel [42,43] and promotes the dynamic ion exchange between the algae and the marine environment through the penetration and diffusion mechanism. The gel network can selectively absorb a variety of metal cations, including Na^+^, Ca^2+^, Mg^2+^, Sr^2+^, Cu^2+^, and Ba^2+^, etc., and sodium alginate powder could be prepared by biosynthesis and industrial extraction and purification [44].

**Figure 2:**
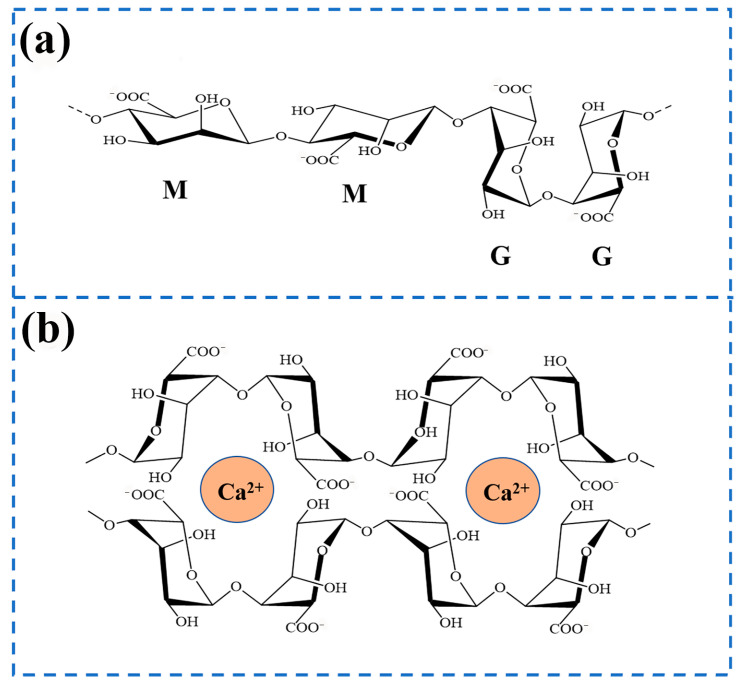
(**a**) Structural formula of sodium alginate. (**b**) Chelation of sodium alginate with Ca^2+^.

SA is a polymer composed of two monomeric units, β-D-glucuronic acid (M) and α-L-guluronic acid (G) [45,46]. Its structural type depends on the different combinations of two monomer blocks, which could be divided into three types: (M-M)_n_, (M-G)_n_, and (G-G)_n_ [47]. The effects of the three combinations are different, as shown in Figure 2a.

MM homopolymer blocks could enhance material flexibility and degradation rate, making them suitable for short-term implant materials. The GG homopolymer block improves the rigidity and stability of gel and is suitable for repairing load-bearing tissues. The MG heteropolymer block serves as a structural transition zone, balancing mechanical properties and biological activity. Different species of brown algae synthesize alginates exhibiting distinct M/G ratios. The M/G ratio represents the proportion of M to G monomers and serves as a key structural parameter for characterizing sodium alginate [48]. The composition of these units fluctuates based on the extraction source, growth environment, maturity level, seasonal changes, and the depth of extraction. Variations in the M/G ratio influence the biocompatibility and biodegradability of alginate biomaterials. The characteristic of being rich in G monomers is generally derived from algal polysaccharides, especially alginate, which endows alginate with unparalleled potential and unique advantages in the biomedical field. In addition, the G monomer sequence in the sodium alginate polymer chain is more inclined to form a cross-linked network with Ca^2+^ and Sr^2+^, etc. [49,50,51,52], through coordination bonds, thus forming a stable eggshell-like structure (as shown in Figure 2b). The gel strength is positively correlated with G content. The G block protonates under acidic conditions with pH < 3, resulting in the contraction of gel, which is suitable for intestinal-targeting drug delivery systems. The negatively charged surface of the G block can adsorb Ca^2+^/PO_4_^3−^ ions, promote the directional deposition of hydroxyapatite, and can be applied to bone tissue engineering scaffolds. However, sodium alginate produced through enzymatic degradation by microorganisms such as nitrogen-fixing bacteria and pseudomonas [53] is often rich in M monomers. Alginate with a higher M/G ratio enhances cell adhesion and proliferation relative to its low-M/G counterpart. It is worth noting that alginates rich in M monomers have attracted much attention due to their unique immunogenicity, which can effectively stimulate the response of the immune system and promote the generation and release of cytokines [54], thus showing broad application prospects in wound healing, tissue scaffolds, and drug delivery.

## 3. Preparation Method of Sodium Alginate Nanofibers

**Figure 3:**
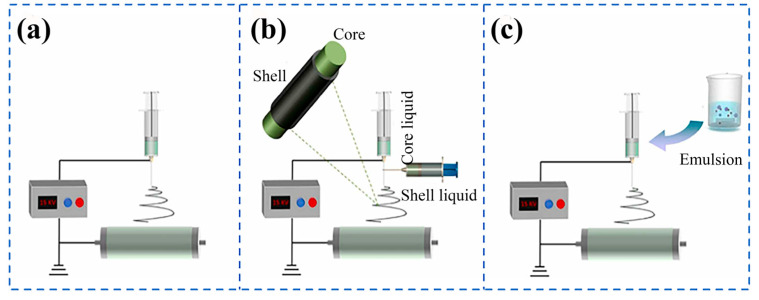
Schematic diagrams of electrospinning classification. (**a**) Blended electrospinning. (**b**) Coaxial electrospinning. (**c**) Emulsion electrospinning [56]. Published by [Elsevier], (2024).

Modern electrospinning technology originated in the 1990s and is an efficient and cost-effective manufacturing process that mainly converts polymer liquid systems into continuous nanoscale fiber structures [55]. The principle is to induce an external electrostatic field, causing the charged polymer fluid to flow out from the nozzle tip, undergo rapid evaporation of the solvent or instant cooling and solidification of the melt, and, finally, deposit on the collection device, forming a fiber network on the nanoscale. SA, as a natural polymer material exhibiting excellent biocompatibility, is environment friendly and renewable, with great potential to promote sustainable development. However, it is difficult to form fibers in its pure solution form. The researchers not only effectively solved the problem of the non-spinnability of the material, but also successfully endowed it with excellent tensile properties and antibacterial properties by blending it with other polymers through blending electrospinning, coaxial electrospinning, and lotion electrospinning [56], as shown in Figure 3. This innovative achievement has widely penetrated into the fields of food [57], sewage treatment [58,59], biomedicine [60], and other multi-functional composite materials [61].

### 3.1. Blended Electrospinning

**Figure 4:**
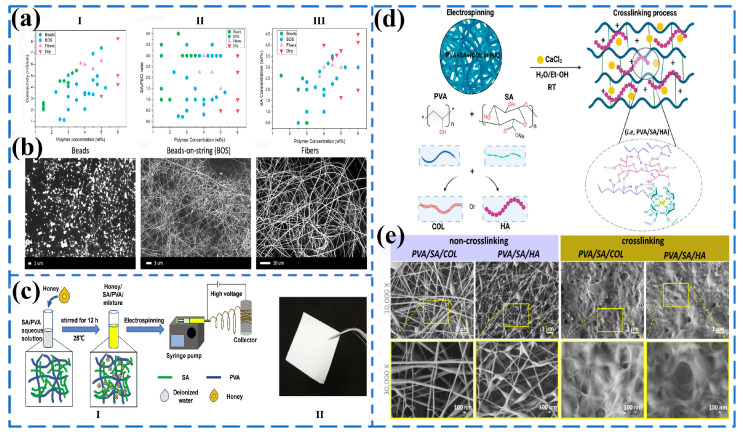
(**a**) Processing regime maps showing the relationship between total polymer concentration and (**I**) conductivity, (**II**) SA:PEO ratio, and (**III**) SA concentration. The narrow window of smooth fiber formation (pink triangles) is highlighted in all maps, but the clearest trend is seen in (**b**). Other morphologies are displayed as beads (green pentagons), beads-on-string (BOS) (blue circles), and drip (red upside down triangles). (**b**) Representative SEM images for each morphology regime [63]. Published by [Springer], (2024). (**c**) Honey/SA/PVA nanofibers fabricated by electrospinning. (**I**) Schematic illustration of the solution preparation and electrospinning process. (**II**) Photograph of honey/SA/PVA nanofiber membrane [67]. Published by [Elsevier], (2019). (**d**) Proposed crosslinking mechanism of the PVA-based scaffolds. (**e**) FE-SEM micrographs displaying the morphology of the mats before (on the left) and after (on the right) being immersed in CaCl_2_ water solution [68]. Published by [Elsevier], (2024).

Blended electrospinning is the process of adding drugs or different solvents to a polymer solution and mixing them evenly before spinning [62]. The properties of additives and polymers could affect the diameter, distribution, and drug release of fibers. PEO, as a synthetic polymer with a linear structure and ether groups, can interact with the hydroxyl groups in sodium alginate to form strong hydrogen bonds, thereby improving the spinnability of sodium alginate. Carroll Bassham et al. [63] systematically analyzed the effect of the interaction between PEO content and surfactants on the diameter of sodium alginate fibers (Figure 4a) and found that, with the decrease in PEO content and the increase in SA concentration, the fiber morphology exhibited a state from beads to fibers and then to droplets, which first increased and then tended to stabilize (Figure 4b).

In addition to PEO [64], PVA [65], gelatin (Gel) [66], and other materials can also be blended with SA to prepare nanofibers at room temperature. Tang et al. [67] incorporated honey into an electrospun nanofiber membrane based on alginate/PVA (Figure 4c), and the average diameter of the nanofibers increased with the increase in honey concentration, which was from 379 ± 65 nm to 528 ± 160 nm. It is because the decrease in the conductivity of the electrospinning solution leads to a reduction in the stretching of the jet, resulting in a larger diameter of the nanofibers. Soto-Quintero et al. [68] studied ion cross-linked PVA/SA fiber scaffolds. During electrospinning, hydrogen bonds were formed between molecules to form a cohesive scaffold structure (Figure 4d). After ion cross-linking, the nanofiber diameters of PVA/SA/collagen (COL) and PVA/SA/hyaluronic acid (HA) scaffolds were 128 ± 35 and 45 ± 22 nm (Figure 4e), respectively. After blending SA with synthetic polymers, spinning is an effective method to improve the physical morphology of SA and enhance fiber properties, greatly reducing the occurrence of fiber adhesion in industrial production processes.

### 3.2. Coaxial Electrospinning

Coaxial electrospinning, also known as dual fluid electrospinning, can prepare nanofibers with optimized core–shell or hollow structures under stable electrostatic fields through a multi-needle system with independent liquid supply [69,70,71,72]. It was first proposed in 2002 [73] and has since been widely developed and applied. The technology can not only achieve synchronous loading of multiple drugs in fibers, but its unique core–shell configuration can also effectively isolate the inner layer drugs and avoid interference from the outer-layer materials, thus demonstrating significant advantages in specific application fields. Wang et al. [74] used microfluidic coaxial electrospinning technology to fabricate core–shell nanofibers by incorporating chlorella pyrenoidosa (CP) extracted by the pH change method into the core and incorporating a combination of SA and PVA into the shell. The co-loading of CP and probiotics improved the thermal performance of nanofibers. The synergistic effect also enhanced their ability to scavenge free radicals while enhancing their antioxidant capacity. The coaxial nanofiber effectively resisted erosion caused by gastric acid and bile salts, improved the survival rate of plant lactobacilli in the gastrointestinal environment, and provided additional protection for probiotics, enhancing their vitality during storage. These findings not only provide a new approach to material preparation for the functional food, nutritional supplements, and biopharmaceutical industries, but also offer valuable insights for the future development of bioactive material carriers. Nista et al. [75] successfully prepared SA-CHI-PEO polyelectrolyte composite nanofiber membranes using coaxial electrospinning technology, with SA/PEO as the core layer solution and chitosan (CHI)/PEO as the shell layer solution. The core–shell fiber membrane has biocompatibility, mucosal adhesion, and structural stability in water, and was expected to serve as a controlled release carrier for oral local administration. Subsequent process optimization was needed to achieve a uniform coaxial structure, further enhancing drug encapsulation and controlled release performance. Hou et al. [76] embedded baicalin into the hydrophobic cavity of hydroxypropyl-β-cyclodextrin through supramolecular assembly to form a complex, and then prepared the complex into a water-soluble antibacterial nanofiber membrane using coaxial electrospinning. In the in vitro release test, nearly 90% of the drug (baicalin) was released from the nanofiber membrane over a period of time, indicating its sustained release ability. In addition, compared with natural baicalin, the complex exhibits higher free radical scavenging and antibacterial rates against *Escherichia coli*, *Staphylococcus aureus*, and *Candida albicans*. It has potential application value in developing wound dressings and drug delivery systems using bioactive compounds extracted from plants.

### 3.3. Lotion Electrospinning

Lotion electrospinning technology is an innovative method for preparing core–shell nanofibers by uniaxial electrospinning based on a stable lotion system [77,78,79]. According to the difference between the dispersed phase and continuous phase, the technology can be divided into two lotion systems: water-in-oil emulsion (W/O) and oil-in-water emulsion (O/W). Under the action of a high-voltage electrostatic field, a biphasic system stabilized by emulsifiers exhibits significant phase separation kinetics during the formation of Taylor cones. This is due to the gradient evaporation effect of solvents between the surface and core layers of the jet, resulting in an exponential increase in the viscosity of the surface solution. Meanwhile, dispersed-phase droplets migrate towards the jet core under the combined drive of interfacial tension and electrorheological effects. During this process, the electric field induced jet stretching and Lorentz force work together to promote the axial alignment of droplets and produce elliptical deformation. Finally, through the continuous evaporation of solvents and the solidification process of polymers, nanofibers with clear core–shell interfaces are formed on the substrate collection device. The significant advantage of this technology lies in its parental drug loading ability, which can simultaneously encapsulate hydrophilic and hydrophobic functional substances and effectively maintain their molecular conformation and biological functionality. The prepared nanofibers not only exhibit excellent sustained release kinetics, but also highlight their practical value due to the simplicity of their preparation process. Negahdari et al. [80] prepared O/W lotion electrospun core–shell mat with polycaprolactone (PCL) as the core and SA and PEO blend solution as the raw materials. The nanofiber demonstrated exceptional cell compatibility and favorable mechanical properties, holding broad potential for biomedical applications. Tao et al. [81] prepared a microfiber biomimetic periosteum of PCL/carboxymethyl chitosan (CMCS)/SA, which had good mechanical properties, good biocompatibility, and good osteogenic induction ability, and could be used as a potential scaffold material for repairing large bone defects.

Compared with the coaxial electrospinning technology, the lotion method has significant advantages in simplifying the process flow and reducing the complexity of equipment and parameter control, which makes it show broad application prospects in the frontier fields such as enzyme immobilization, intelligent drug delivery systems, functional wound dressings, and active food packaging films [82,83]. However, lotion electrospinning also presents limitations, including relatively poor stability and the inability to use low-surface-tension polymer solutions. Lotions exhibit inherent susceptibility to destabilization over time, necessitating the incorporation of stabilizers such as emulsifiers to enhance stability [84]. In addition, the advantages and disadvantages of different electrospinning methods are summarized in Table 1.

## 4. Application of Sodium Alginate Composite Nanofibers in Biomedical Field

### 4.1. Tissue Engineering Scaffolds

The conventional treatment methods for tissue or organ injuries, such as autologous transplantation, allogeneic transplantation, and artificial prosthesis implantation, have problems such as donor shortage, immune rejection, and the risk of secondary surgery [86]. For example, autologous transplantation may cause complications in the donor site, allogeneic transplantation requires the long-term use of immunosuppressive agents, and artificial prostheses cannot achieve biological integration. With the development of stem cell technology, biomaterials science, and three-dimensional (3D) printing [87], regenerative medicine has become a new direction for solving tissue defects. As a core carrier, tissue engineering scaffolds simulate the ECM to provide a growth microenvironment for cells and guide tissue regeneration [88,89]. The emergence of new biomaterials such as PVA, SA, and polylactic acid (PLA), etc. has enabled scaffolds to have adjustable porosity, degradation rate, and mechanical strength, meeting the repair needs of different tissues. The following is mainly summarized regarding three aspects: skin, gums, and heart.

**Figure 5:**
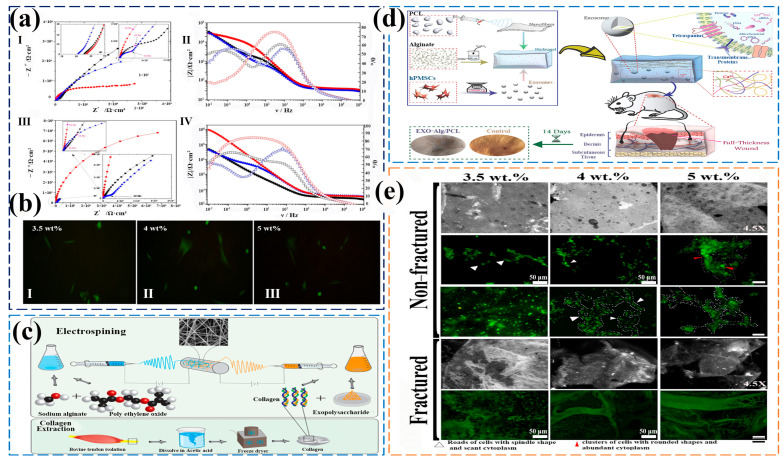
(**a**) Nyquist and Bode plots for PVA/SA samples with and without the incorporation of HGF after 72 h of immersion in biological solution at 35 °C in a CO_2_ atmosphere. (**I**) Nyquist plot for systems without the incorporation of HGF (where Z″ is the imaginary part and Z′ the real part of the complex impedance); (**II**) Bode plot for systems without the incorporation of HGF; (**III**) Nyquist plot for systems with the incorporation of HGF; (**IV**) Bode plot for systems with the incorporation of HGF [93]. The datasets values (colour, shape, size) are the same for all the graphs (3.5%wt ■, 4.0%wt 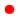 and 5.0%wt 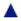, respectively). In Bode plots the filled symbols represent the impedance modulus, |Z| and the non-filled ones represent the phase shift, θ, against frequency, υ. (**b**) Fluorescence assessments of HGF systems (**I**) 3.5 wt%, (**II**) 4 wt%, and (**III**) 5 wt% after 24 incubation time [93]. Published by [Elsevier], (2020). (**c**) Fabrication process of biaxially electrospun collagen/alginate nanofibers, improved with *Rhodotorula mucilaginosa* sp. GUMS16-produced exopolysaccharides for wound healing applications [92]. Published by [Elsevier], (2022). (**d**) Fabrication procedure of multifunctional hybrid scaffolds loaded with exosomes, and their application for the healing of a full-thickness wound in a rat model [91]. Published by [Elsevier], (2025) (**e**) Scaffold architecture/cell seeding/viability on PVA/SA scaffolds [94]. Published by [Elsevier], (2024).

The skin is an essential organ for maintaining life and health. Tissue engineering scaffolds can load various bioactive molecules, such as growth factors, antibiotics, and anti-inflammatory drugs, etc., and continue to be released locally in the wound to create an optimal healing microenvironment. Sasan et al. [90] prepared an innovative porous nanofiber membrane as a dressing patch with antibacterial and anti-inflammatory functions for skin wound healing. ZnO NPs and Salvia miltiorrhiza essential oil (SAEO) were incorporated into SA, with SA serving as the outer shell and ε-polycaprolactone as the core of coaxial electrospun wound dressing nanofibers. The average diameter of the nanofibers is 187 ± 51 nm, with a tensile strength of 4.7 ± 0.4 MPa, an elongation at break of 32.9 ± 2.1, and an elastic modulus of 21.4 ± 2.0. The simultaneous application of ZnO NPs and SAEO significantly enhanced antibacterial activity against Staphylococcus aureus and Escherichia coli, while promoting the proliferation, attachment, and viability of L929 cells with viability exceeding 90%, thus establishing its potential as an ideal wound dressing material for full-thickness wound repair. Owing to its ECM-mimicking porous structure, bionic electrospun nanofibers form a hybrid system integrating hydrophilic hydrogel with hydrophobic electrospun matrices. This architecture establishes an effective platform for biomolecular delivery and enhanced wound tissue regeneration. Ashrafi et al. [91] prepared a multifunctional hybrid scaffold of hydrogel electrospun nanofiber, and evaluated the effectiveness of the hybrid design of nanofiber and hydrogel in controlling the delivery of exosomes (EXOs) to the wound to enhance the healing process (Figure 5d). The research results indicated that the stent loaded with EXOs accelerated wound closure. Histological studies on the 14th day of treatment showed granulation tissue formation, reepithelialization, and hair follicle growth. Through EXOs-Alg/PCL synthesis, up to 65% of type I collagen formation and up to 98% of epithelial re-formation were achieved, which has certain potential in promoting tissue growth and healing. Although the proposed bioactive design demonstrated potential for enhancing exosome delivery to accelerate wound healing, its clinical significance and safety concerns warrant further examination. Ashraf et al. [92] used bidirectional electrospinning technology to prepare a tissue engineering scaffold made of COL/SA/PEO/extracellular polysaccharides (EPS) from red yeast (Figure 5c). COL-SA/PEO+EPS cross-linked nanofibers exhibit over 100% cell viability. In addition, scanning electron microscopy images of cells on electrospun scaffolds showed that all nanofibers had good cell growth and proliferation abilities, indicating that EPS produced by Rhodotorula mucilaginosa sp. GUMS 16 in electrospun fibers could be considered a novel biomacromolecule that could improve cell survival and proliferation.

The ideal scaffold not only contributes to gum cell growth, but also provides a suitable microenvironment for tissue regeneration. Hernández et al. [94] deposited PVA/SA solutions of different concentrations on the surface of electrode microchips using electrospinning technology to form nanofiber scaffolds. The scaffold exhibited the best biocompatibility at the optimal concentration of human gingival fibroblast (HGF) (Figure 5e), with high resistance that is 1 × 10^6^ Ω and low capacitance that is 1 μF indicating stable deep cell adhesion and high biofilm maturity. It provided new ideas for the design of tissue engineering scaffolds, indicating that the biocompatibility of scaffolds can be optimized by regulating material concentration and electrospinning processes, promoting their application in periodontal tissue regeneration and other fields. The fusion of gingival cells with appropriate biological scaffolding substances represents a significant research avenue in the fields of tissue engineering and regenerative medicine. In addition, Hernández’s other teams [93] used electrospinning technology to prepare PVA/SA composite scaffold materials for the cultivation of rat cardiomyocytes. The results indicated that the morphology and proliferation rate of cells depended on their growth rate, and a 5.0% by weight scaffold has been proven to be the most effective as it produced the highest impedance amplitude and the highest number of cells, with more organized network formation (Figure 5a,b). In summary, electrospinning combined with bioactive factor incorporation generated a favorable porous microenvironment for cells, significantly enhancing viability and facilitating regeneration.

### 4.2. Wound Dressings

**Figure 6:**
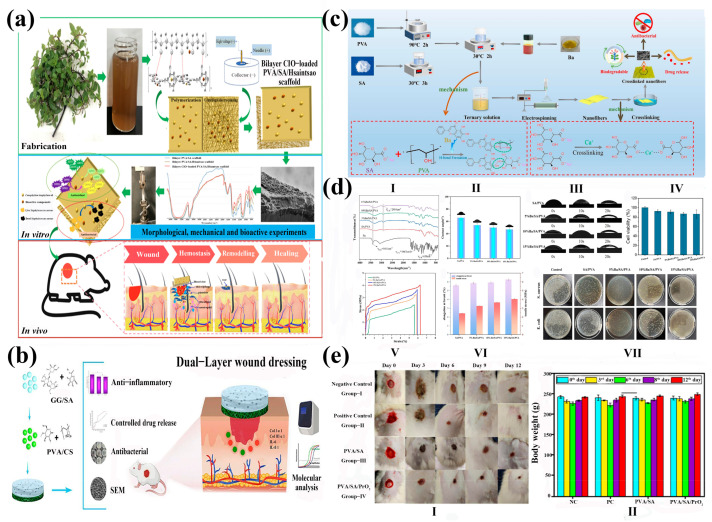
(**a**) A general diagram of the study representing the fabrication of the bilayer CIO-loaded PVA/SA/Hsiantsao scaffold, analysis of morphological, mechanical, biochemical, and biocompatible characterizations in vitro, and its mechanism in wound healing in vivo [99]. Published by [Elsevier], (2025). (**b**) Preparation and related tests of dual-layer alginate hydrogel dressings with chitosan nanofibers [100]. Published by [Elsevier], (2025). (**c**) Preparation process and mechanism of wound dressing [101]. (**d**) (**I**) FTIR spectra of nanofibers; (**II**,**III**) water contact angle of nanofibers; (**IV**) cell viability of nanofibers; (**V**,**VI**) mechanical property of nanofibers; (**VII**) antibacterial activity of nanofibers [101]. Published by [Elsevier], (2025). (**e**) (**I**) Wound healing in rats. (**II**) Body weight analysis of the experimental animals; ns represents no significance [98]. Published by [Elsevier], (2024).

As a critical biomedical implementation of electrospun nanofibers, wound dressings fulfill multifunctional imperatives, mitigating nociceptive responses, conferring antimicrobial efficacy, and accelerating tissue regeneration [95,96], while concurrently establishing a protective barrier against exogenous environmental insults. Leveraging its inherent biocompatibility, sodium alginate demonstrates significant utility in both stenting applications and advanced wound matrices, where it potentiates therapeutic outcomes through synergistic drug delivery paradigms. Mavrokefalou et al. [97] prepared two types of functionalized nanofiber membranes using electrospinning technology. Both systems were based on SA, PEO, and betamethasone (Beta) with anti-inflammatory activity, endowing the materials with wound healing and anti-inflammatory properties, respectively. One of the systems additionally introduced Gel as a variable to evaluate its potential synergistic effect on enhancing wound healing performance. The research showed that the nanofibers containing SA could shorten the BCT time by 1/3, which indicated that SA has better hemostatic properties in vitro than gel, showing great potential for wound healing research. Natural polymer materials are highly favored in wound dressings due to their biodegradability, absorbency, biocompatibility, and good mechanical properties. SA, a naturally derived polysaccharide, exhibits exceptional hydrophilicity and moisture retention capacity. This biomaterial facilitates the significant absorption of wound exudates, enhances epidermal regeneration kinetics, and mediates the sustained release of antimicrobial/anti-inflammatory agents, establishing its utility as a premium medical dressing. Hiytesh et al. [98] successfully prepared PVA/SA/Praseodymium oxide (PrO_2_) nanofibers using electrospinning, with a diameter significantly smaller than PVA/SA fibers. The nanoscale PrO_2_ exhibited antibacterial activity against wound pathogens, showing 60%, 50%, 33%, and 41% bacterial growth inhibition against *Escherichia coli*, *Pseudomonas aeruginosa*, and *Escherichia faecalis* respectively. PVA/SA/PrO_2_ fibers had good biocompatibility and could promote blood coagulation, cell growth, and migration. PVA/SA/PrO_2_ fibers showed better wound healing effects in in vivo experiments, with a wound closure rate of 95% (Figure 6e), confirming the potential use of PVA/SA/PrO_2_ fibers in wound dressings.

Hydrogels, characterized by ultrahigh water content (>80%), replicate native tissue microenvironments through biomimetic extracellular matrix architectures [102]. Their intrinsic permeability facilitates bidirectional diffusion of oxygen, nutrients, and metabolic wastes, driving the development of electrospun bilayer wound dressings with synergistic functionality. Abdollahi et al. [100] engineered a bilayer wound dressing system through electrospinning composite technology, utilizing sodium alginate–guar gum as the foundational matrix (Figure 6b). The inner stratum consisted of this alginate–guar complex, while the functional encapsulation layer constituted ciprofloxacin-enriched polyvinyl alcohol–chitosan nanofibers. Quantitative analysis revealed a hemolysis rate of 1.13 ± 0.03% for hydrogel nanofibers, confirming outstanding hemocompatibility. Furthermore, the bilayer architecture significantly accelerated wound re-epithelialization in murine models, manifesting as substantial wound contraction and enhanced collagen deposition density. The decrease in IL-1 β and IL-6 that is *p* < 0.05 confirms the efficacy of the membrane in wound healing and inflammation relief. Luong et al. [99] prepared a double-layer (*Calophyllum inophyllum* seed oil) CIO-loaded PVA/SA/Hsiantsao as a wound dressing. The scaffold, composed of a semi-hydrophobic hydrogel matrix and hydrophilic nanofibers, was fabricated through synergistic polymerization and centrifugal electrospinning techniques (Figure 6a). The results showed that the manufactured bilayer material exhibited good biocompatibility and strong antioxidant and antimicrobial activity, with a DPPH clearance rate of 82.19% ± 0.08 and an ABTS clearance rate of 90.23% ± 0.22. In a rat wound model, PVA/SA/Hsiantsao scaffolds loaded with CIO significantly improved wound healing on day 15, with a wound healing rate of 98.22% ± 0.82. The in vitro degradation rate reached as high as 47% within one month, demonstrating its ecological friendliness. Qi et al. [101] developed a SA/PVA composite medical dressing containing baicalein (Ba) using electrospinning technology (Figure 6c). The in vitro experimental results showed that the antibacterial rates of 15% Ba/SA/PVA composite nanofibers against Staphylococcus aureus and Escherichia coli were 98.86% and 99.58%, respectively, indicating that it is a wound dressing with good antibacterial properties (Figure 6d). Traditional medical dressings are usually made of freeze-dried sponge or non-woven microfibers, while nanofiber matrices have porosity, excellent oxygen permeability, and easily adjustable mechanical strength, making them closer to native tissues. Hajiali et al. [103] treated sodium alginate nanofibers with trifluoroacetic acid (TFA) to generate insoluble poly (alginic acid), which significantly improved the stability of the nanofibers in pH 7.4 buffer solution. The diameter of the nanofibers was approximately 90 ± 20 nm. Depending on the duration of TFA treatment, their degradation time extended from 7 days to 14 days, and their cell compatibility was good.

### 4.3. Drug Delivery

**Figure 7:**
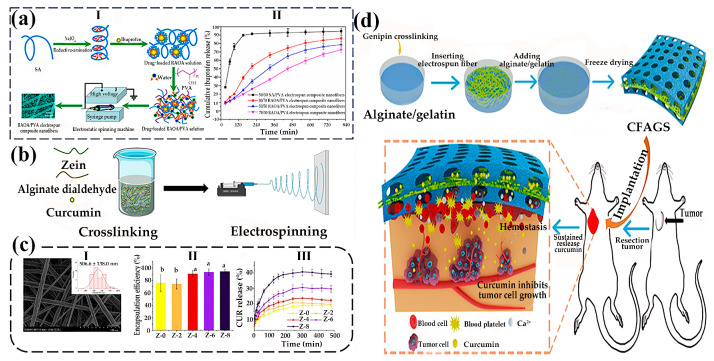
(**a**) (**I**) Schematic diagram of fabrication of drug-loaded RAOA/PVA electrospun composite nanofibers; (**II**) Release of ibuprofen from 50/50 SA/PVA, 30/70 RAOA/PVA, 50/50 RAOA/PVA and 70/30 RAOA/PVA electrospun composite nanofibers in pH 7.4 PBS medium at 37 °C (error bars represent the standard deviation of three replicates) [105]. Published by [Elsevier], (2022). (**b**) Preparation scheme of dialdehyde alginate cross-linked on curcumin-laden zein gliadin nanofibers. (**c**) (**I**) FE-SEM micrographs and fiber diameters of electrospun fibers for Z-8; (**II**) The encapsulation efficiency of the fibers; (**III**) The CUR release profiles of the fibers. Different letters indicated significant differences (*p* < 0.05) [106]. Published by [Elsevier], (2024). (**d**) Schematic illustration of a novel alginate/gelatin sponge combined with curcumin-loaded electrospun fibers for postoperative rapid hemostasis and prevention of tumor recurrence [107]. Published by [Elsevier], (2021).

Sustained release is a sustained and slow drug release process. In order to achieve safe, effective, long-lasting, and convenient drug release, it can also be called sustained drug release (SDR). The development of a complex structure electrospun nanofiber process for the one-step production of multi-chamber fibers has received widespread attention as a new strategy for developing novel SDR nanomaterial platforms [104]. Due to the biocompatibility of SA and the controllability of electrospun fibers, drugs can be loaded through a combination of modification and blending, achieving long-lasting or even sustained drug release for several days or even months. Chen et al. [105] successfully synthesized the reductive amination of oxidized alginate derivative (RAOA) from SA through redox amination reaction, and prepared RAOA/PVA electrospun composite nanofibers using electrospinning (Figure 7a). The oxidation–reduction amination reaction was used to repair SA breaks its intramolecular hydrogen bonds, which is beneficial for enhancing the molecular flexibility and colloidal interfacial activity of RAOA. RAOA had a good affinity for hydrophobic ibuprofen, achieving loading and controlled release of hydrophobic drugs. The rapid release of ibuprofen in unmodified SA/PVA (50/50) was achieved by electrospinning fibers at 37 °C in pH 7.4 PBS medium, with a release rate of about 90%. By changing and adjusting the polymer ratio, the drug release time could be significantly increased to 810 min due to the enhanced solubility and encapsulation properties of the polymer. In addition, RAOA/PVA electrospun composite nanofibers had low cytotoxicity towards L929 cells, allowing the drug to maintain a stable concentration at the wound site for a longer period of time, thereby improving drug utilization and sustainably promoting wound healing. Li et al. [106] modified sodium alginate and subsequently prepared electrospun zein/alginate formaldehyde (AD) nanofibers through green cross-linking (Figure 7b). The cross-linking degree could reach 50.72%, and the diameter range of electrospun fibers was 446.2 to 541.8 nm (Figure 7c). The embedding efficiency of Z-0 was 75.73%, and the addition of 2% AD (74.11%) had no significant effect on the efficiency. When the concentration of AD reached 4%, the embedding efficiency was significantly improved, and the values of Z-4, Z-6, and Z-8 were 90.36%, 92.91%, and 93.89%, respectively, which were statistically the same. The high embedding efficiency of highly cross-linked fibers was related to a denser fiber network, which would be beneficial for protecting the loaded bioactive substances. The incorporation of AD enhanced the elasticity of the fibrous membrane, concurrently improving the encapsulation efficiency and sustained release performance, demonstrating its potential for drug delivery applications.

Electrospun nanofiber systems enable dual-functional payload delivery: the matrix encapsulation of therapeutic agents via blending techniques and probiotic encapsulation through coaxial electrospinning. This architecture mediates targeted in vivo release kinetics, facilitating the precision modulation of gastrointestinal microbiome homeostasis and associated physiological outcomes. Diep et al. [108] blended the probiotic Lactococcus with SA into a core solution, with SA/PEO/polysorbate 80 (PS80)/CaCO_3_ as the shell solution, and encapsulated the probiotic Lactococcus probiotic into alginate-based nanofibers containing acid-fast calcium carbonate by coaxial electrospinning. High-molecular-weight polyethylene oxide was used to promote fiber cross-linking and protect embedded live bacteria from acid damage with antacids. After dissolving in water, polyethylene oxide diffused out of the nanofibers to form a safe oral formula. Without antacids, no viable bacteria were detected in the simulated gastrointestinal solution. It proved that preparing 2% calcium carbonate (antacid) into an external precursor solution is crucial for the survival of embedded lactobacilli in the simulated gastrointestinal tract. Adding antacids would not affect the bacterial load within the nanofibers. Therefore, bacteria remained active and enveloped during immersion in the simulated stomach model. After transferring to the intestinal phase, up to 120,000 live probiotic cells were released per gram of nanofibers, demonstrating the pH-dependent delivery of electrospun algal nanofibers. Chen et al. [107] prepared a novel alginate/gelatin sponge combined with curcumin-supported electrospun fibers (CFAGS) to rapidly stop bleeding and prevent tumor recurrence by employing an electrospinning interosmotic polymer network (IPN) strategy (Figure 7d). The results demonstrated that the alginate/gelatin sponge exhibited superior hemostatic efficacy compared to commercial gelatin-based hemostatic sponges. Notably, CFAGS facilitated sustained curcumin release and promoted its accumulation at tumor resection sites, thereby inhibiting local tumor recurrence in a postoperative subcutaneous model. In summary, sodium alginate-based electrospun nanofibers demonstrated certain potential in modulating the gastrointestinal microenvironment and suppressing tumor recurrence through active drug targeting, indicating considerable promise for biomedical applications.

## 5. Conclusions and Future Perspectives

SA is a natural anionic polysaccharide with abundant and non-toxic raw materials, which has high potential for application in the field of biomedicine. Pure SA solution is difficult to spin. By employing blending modification and functional modification, the electrospinnability of SA is enhanced to prepare composite nanofibers. These modifications endow the material with integrated multifunctional properties which include antibacterial activity, antioxidant capacity, and pH/ion-responsive drug release. This strategy significantly enhances chronic wound repair outcomes. In addition, its biocompatibility and degradability avoid the risk of secondary surgery, and its mechanical properties could be optimized by regulating the M/G block ratio. Using electrospinning technology to fiberize sodium alginate, its high specific surface area and high porosity endow it with extracellular matrixlike properties, which could promote cell adhesion and proliferation. However, the current application of SA electrospinning nanofibers still faces some challenges. On the one hand, the rigid chain of natural sodium alginate leads to poor spinnability, requiring the use of synthetic polymer blending or chemical modification to improve fiber formation, which may introduce biological toxicity risks. This risk can be avoided by using biocompatible polymers (e.g., PVA) or other drugs. On the other hand, SA electrospinning has relatively low productivity, which may be solved by modifying the structural aspects of the electrospinning setup (e.g., multi-needle arrangement). While SA electrospun nanofibers are in large-scale production, the precise control of process parameters such as voltage and solution viscosity, as well as the long-term biological safety evaluation of in vivo testing, still require breakthrough. Future research should consider (1) the methods for electrospinning derivatives of alginate without the help of carrier polymers and/or co-solvents and (2) that, through cross-field combination, clinical transformation is achieved.

In conclusion, sodium alginate holds great promise in the field of biomedicine. SA will further combine technologies in other fields to build multifunctional composite materials through molecular weight regulation and process optimization, which will promote its large-scale application in the fields of tissue engineering stents, wound dressings, and drug release, and contribute to human health and a happy life.

## Figures and Tables

**Table 1:** Advantages and disadvantages of three methods of electrospinning.

Method	Advantages	Disadvantages
Blended electrospinning	One-step molding, easy to process, low equipment requirements [60]	Limited functionality, easy to disperse unevenly, and initial burst release [60]
Coaxial electrospinning	Core–shell structure nanofibers can be prepared, functional partitioning can be implemented, sustained release of the drug can be achieved [74,75]	Low efficiency, complex operation, and high requirement of process variables [78]
Emulsion electrospinning	The core–shell structure can be prepared on a single axis, and it can contain both hydrophilic and lipophilic drugs, simplifying the process flow and reducing the complexity of equipment and parameter control [84]	Emulsion instability, relatively low productivity [85]

## Data Availability

No new data were created or analyzed in this study. Data sharing is not applicable to this article.

## Abbreviations

The following abbreviations are used in this manuscript:

SA	Sodium alginate
PVA	Polyvinyl alcohol
PEO	Polyethylene oxide
ECM	Extracellular matrix
Dex	Dexpanthenol
ZnO NP	Zinc oxide nanoparticles
PLCL	Poly(L-propylcaprolactone CO)-ε-caprolactone)
Gel	Gelatin
COL	Collagen
HA	Hyaluronic acid
CP	Chlorella pyrenoidosa
CHI	Chitosan
W/O	Water-in-oil emulsion
O/W	Oil-in-water emulsion
PCL	Polycaprolactone
CMCS	Carboxymethyl chitosan
3D	Three-dimensional
HGF	Human gingival fibroblast
SAEO	Salvia miltiorrhiza essential oil
EXOs	Exosomes
EPS	Extracellular polysaccharides
Bate	Betamethasone
PrO_2_	Praseodymium oxide
CIO	Calophyllum inophyllum seed oil
DPPH	2,2-diphenyl-2-picrylhydrazyl
ABTS	2,2-azino-bis-3-ethylbenzothiazoline-6-sulfonic acid
Ba	Baicalein
TFA	Trifluoroacetic acid
SDR	Sustained drug release
RAOA	Reductive amination of oxidized alginate derivative
PBS	Phosphate buffer saline
AD	Alginate formaldehyde
PS80	Polysorbate
CaCO_3_	Calcium carbonate
PDLGA	Poly(D,L-lactide-co-glycolide)
PCU	Polycarbonate polyurethane
CFAGS	A novel alginate/gelatin sponge combined with curcumin-supported electrospun fibers
IPN	Interosmotic polymer network
